# First continental troglobiont *Cylindroiulus* millipede (Diplopoda, Julida, Julidae)

**DOI:** 10.3897/zookeys.795.27619

**Published:** 2018-11-08

**Authors:** Ana Sofia P.S. Reboleira, Henrik Enghoff

**Affiliations:** 1 Natural History Museum of Denmark, University of Copenhagen, Universitetsparken 15, DK-2100 København Ø, Denmark University of Copenhagen København Denmark

**Keywords:** cave fauna, Julida, karst, Portugal, troglobiont

## Abstract

The new species of millipede *Cylindroiulusvillumi* is described from a cave in the Estremenho karst massif in central Portugal. It is the first cave-adapted species of its genus with a strict subterranean life-style in continental Europe, and is the fifth blind species of the genus. The new species is illustrated with photographs and diagrammatic drawings. It is tentatively placed in the purely Iberian *Cylindroiulusperforatus*-group. The differences between the new species and its relatives are discussed, as well as its adaptations to a subterranean life-style.

## Introduction

The genus *Cylindroiulus* Verhoeff, 1894 belongs to the Palaeartic family Julidae and has more than 100 species distributed in Macaronesia and Europe (Kime and Enghoff 2017), North Africa (Read 2005, Akkari and Enghoff 2008), Turkey, the Caucasus region and Iran (Read 1992), and Central Asia (Read 1994). The genus can be recognized by the lack of frontal and metazonital setae, expanded mandibular stipites in males, gonopods with a flagella, and free, unforked mesomerites separated by a deep and wide incision from the opisthomerite (Read 1990).

Cave-adapted species of *Cylindroiulus* species were only known from Madeira Island, whereas in continental Europe only two anophthalmic, but not troglobiont, species were known (Reboleira and Enghoff 2014a, Read 2007).

Nine species of *Cylindroiulus* are currently known from mainland Portugal: *C.anglilectus* Read, 2007, *C.boreoibericus* Read, 2007, *C.britannicus* (Verhoeff, 1891), *C.caeruleocinctus* (Wood, 1864), *C.fenestratus* Read, 1989, *C.latestriatus* (Curtis, 1845), *C.perforatus* Verhoeff, 1905, *C.propinquus* (Porat, 1870), and *C.truncorum* (Silvestri, 1896) (Read 2007, Kime and Enghoff 2017).

Only very recently cave-adapted species of millipedes from Portugal have started to dig out of the dark and become known to science (Enghoff and Reboleira 2013, Reboleira and Enghoff 2013a, b, 2014a, b, c, 2017). Recently, a new species of *Cylindroiulus* has been found in a cave in the Estremenho massif in central Portugal. This is the largest karst area of Portugal, mainly composed of Jurassic limestone; a considerable part of its area is included in the Serra de Aire e Candeeiros Natural Park. We here describe this first cave-adapted species of *Cylindroiulus* from the European continent.

## Materials and methods

Sampling was performed by direct search in the cave Algar do Pena in Estremenho karst massif, central Portugal.

Specimens were examined under a binocular stereomicroscope Leica M165C, and measurements were made with the software Leica Application Suite V4.12. Gonopods, vulvae, legs and antennae were dissected and mounted on temporary slides in glycerine or lactic acid for study under light microscopy in a Leica DM2500 microscope. Measurements were made following the methodology described by Enghoff (1982). Images of the gonopods and vulvae were stacked with the software Zerene Stacker. For scanning electron microscopy (SEM) one head, gonopod, leg, and tail were mounted on aluminium stubs, coated for 110 seconds with platinum/palladium, and studied in a JEOL JSM-6335F microscope. The background of some SEM images was processed with Adobe Photoshop CS6.

The type material is deposited in the collection of the Natural History Museum of Denmark, University of Copenhagen (**NHMD**, formerly ZMUC).

## Results

### Order Julida Brandt, 1833

#### Family Julidae Leach, 1814

##### Genus *Cylindroiulus* Verhoeff, 1894

###### Cylindroiulus villumi sp. n.

Taxon classificationAnimaliaJulidaJulidae

http://zoobank.org/79A57B30-7ABF-4FCB-9B94-CB0A459DB129

**Figure 1.**
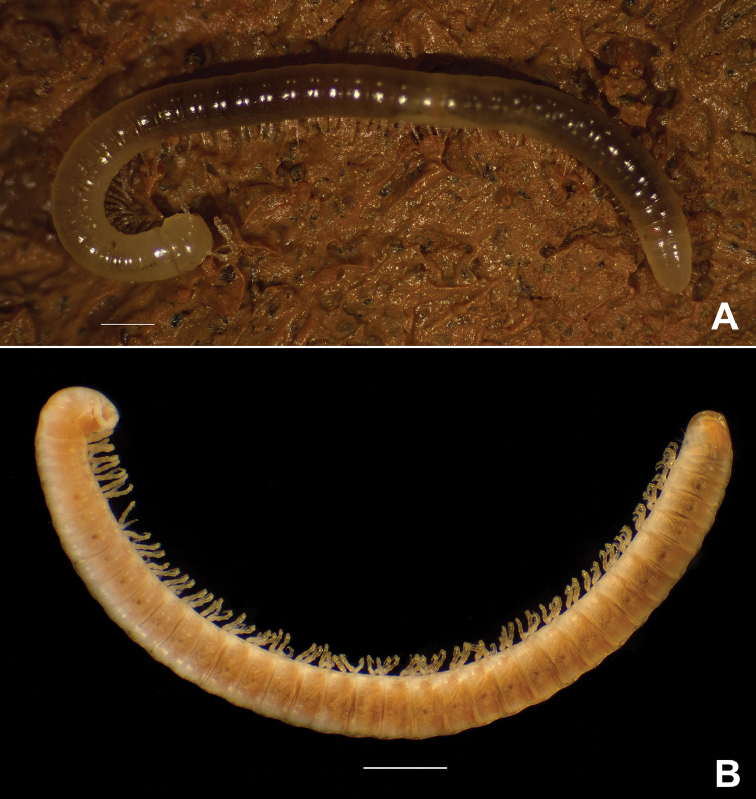
*Cylindroiulusvillumi* sp. n. **A** habitus of live female **B** habitus of subadult male. The partly darker colouration in 1B is due to gut contents seen by transparency. Scale bar: 1 mm.

**Figure 2.**
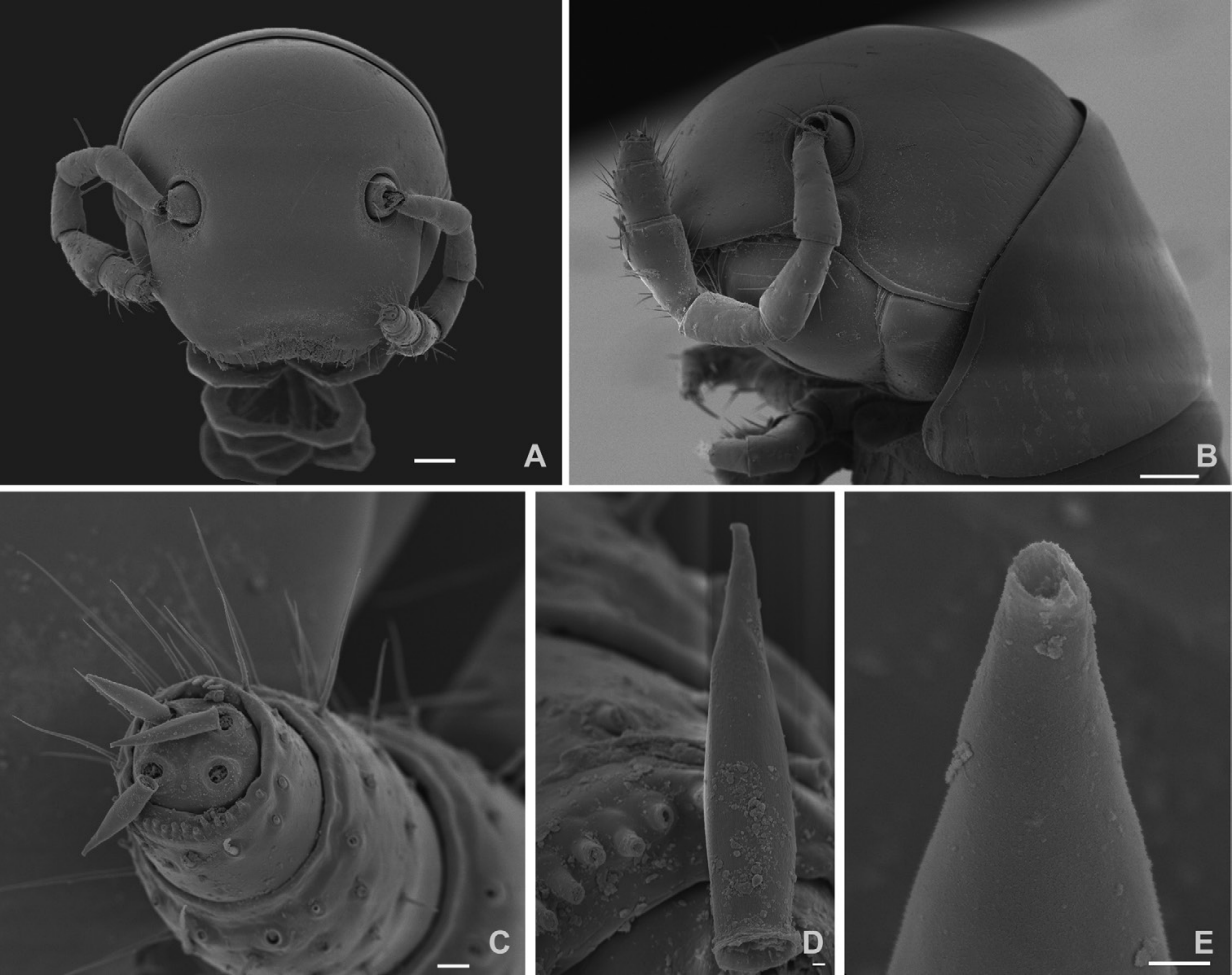
*Cylindroiulusvillumi* sp. n. female paratype, SEM. **A** anterior view of the head **B** lateral view of the head **C** tip of the antenna **D** detail of a sensory cone of the antenna **E** tip of the sensory cone. Scale bars: 100 μm (**A, B**); 10 μm (**C**); 1 μm (**D, E**).

**Figure 3.**
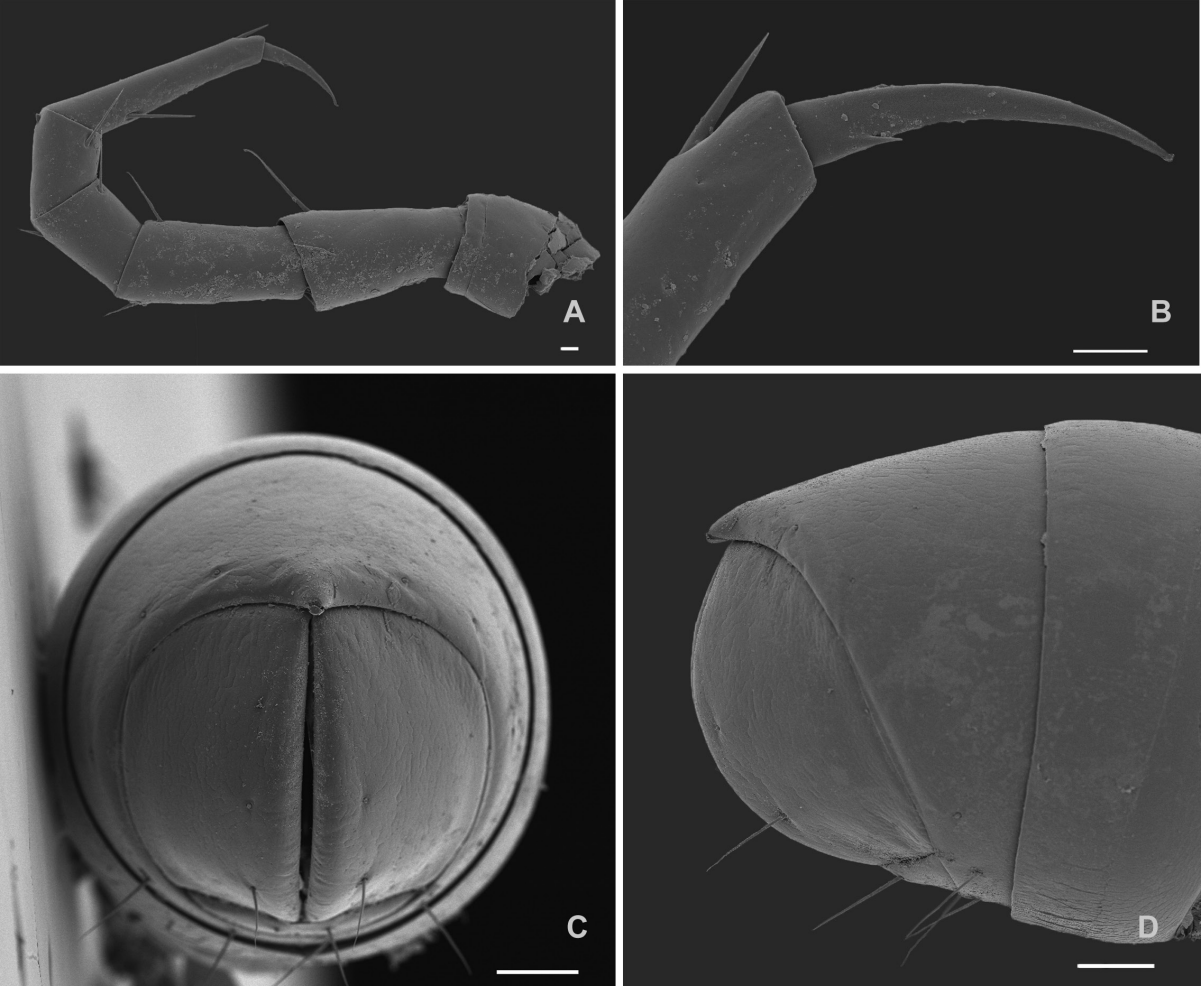
*Cylindroiulusvillumi* sp. n. female paratype, SEM. **A** midbody leg **B** detail of the claw **C** posterior view of the anal valves **D** lateral view of the telson. Scale bars: 10 μm (**A, B**); 100 μm (**C, D**).

**Figure 4.**
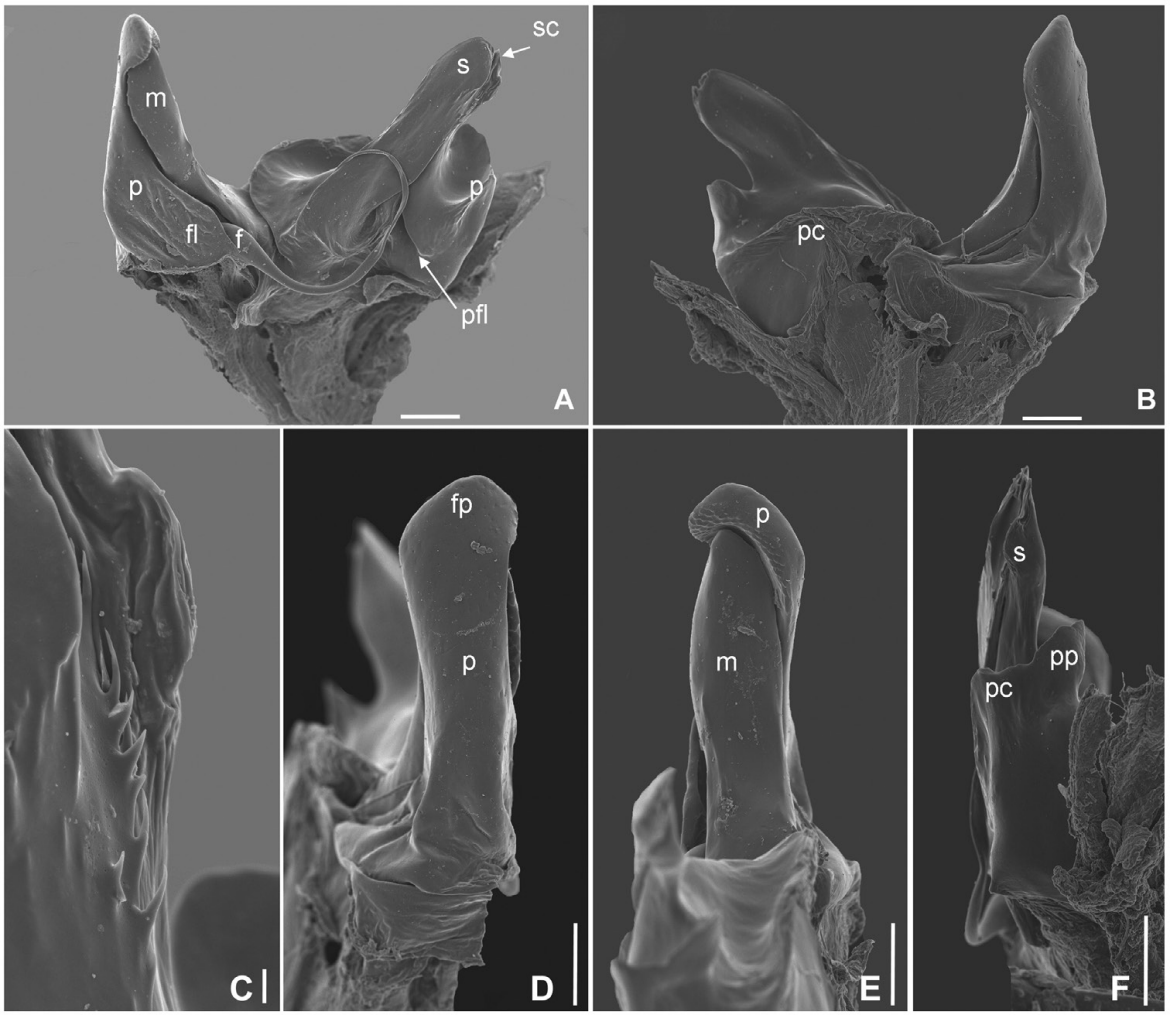
*Cylindroiulusvillumi* sp. n. SEM of the male gonopod. **A** mesal view **B** lateral view **C** denticles on the anterior flagellum-conducting lamella of the solenomerite **D** pro- and mesomerite, anterior view **E** pro- and mesomerite, posterior view **F** opisthomerite, posterior view. Abbreviations: f: flagellum, fl: flagelliferous lobe of promerite, fp: finger-shapped projection of promerite, m: mesomerite, p: promerite, pc: lateral rim of paracoxite, pfl: posterior flagellum-conducting lamella, pp: paracoxal process, s: solenomerite, sc: sperm canal. Scale bars: 10 μm (**A, B, D, E, F**); 1 μm (**C**).

**Figure 5.**
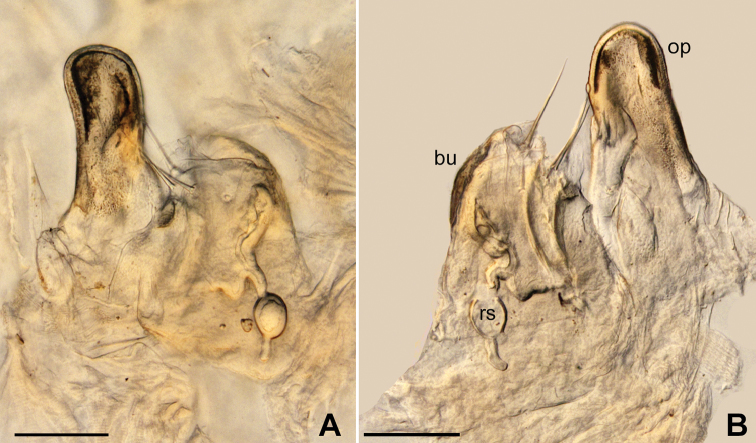
*Cylindroiulusvillumi* sp. n. vulva, lateral view. Abbreviations: bu: bursa, op: operculum, rs: receptaculum seminis. Scale bar: 100 μm.

Figs 1 , 2 , 3 , 4 , 5

####### Type material.

**Holotype**, male, Portugal, Estremenho karst massif, Algar do Pena Cave (Coordinates: 39°27'54.40"N, 8°48'25.24"W), ASPS Reboleira leg., 04 Nov 2014. **Paratypes**: Portugal, Estremenho karst massif, Algar do Pena Cave, ASPS Reboleira leg., 04 Nov 2014, 1 male, 2 females, 4 juvenile males and 1 juvenile; same data but 28 Mar 2018, 1 female and 1 juvenile.

####### Diagnosis.

A medium to small, blind, and unpigmented species of the *Cylindroiulusperforatus*-group. Anterior constriction pronounced and pilosity of the telson scarce. Differs from all other species in the group by the lack of eyes and by the shape of the gonopod mesomerite which is shorter than the promerite (>< *C.fenestratus* Read, 1989, *C.perforatus* Verhoeff, 1905, and *C.ventanaea* Read, 2007) and apically rounded (>< *C.anglilectus* Read, 2007). Further differs from other group members except *C.anglilectus* by the much shorter paracoxal process.

####### Description.

Male holotype: 37 podous + 1 apodous rings + telson; females up to 41 podous + 1 apodous rings + telson.

*Body length* up to 13 mm in females and 11.4 mm in males. Vertical body diameter (H): 0.9 mm (females) and 0.7 mm (males). Integument unpigmented (Figure 1); eyes absent (Figs 1, 2A, B). Length of antennae 0.8 mm (Figure 2B), with sensory cones elongated and with a fine longitudinal striation (Figure 2C, D) ending in a pore as shown in Figure 2E. Anterior constriction of body pronounced in dorsal view. Limbus of the the normal type *sensu*Enghoff (1982), i.e., with simple marginal cells without denticles on the free margin. Length of legs (Figure 3A) 1.8 mm, tarsus being the longest podomere. Length of claw 9.6% of total leg length. Accessory claw exceptionally short: 92% shorter than the claw (Figure 3B). Preanal ring with a very short blunt projection, almost glabrous, only with 5 lateral setae (Figure 3C, D), subanal scale with two setae, anal valves with two long ventral setae on the lateral part of the posterior margin, rarely up to two additional setae were observed, however the number is variable and may even differ between right and left valve of the same specimen (Figure 3C). Male first pair of legs modified as typical of the genus, hook-like.

*Gonopods* (Figure 4): Promerite in anterior view (Figure 4D), higher than mesomerite (Figure 4D, E), with rugose area facing apical part of the mesomerite (Figure 4E). Mesomerite (Figure 4E): slender, shorter than, and fitting into, apical concavity of promerite. Paracoxal rim moderately developed. Paracoxal process not very prominent, rather short and mostly fused to solenomerite (Figure 4F). Solenomerite as in Figure 4A, B, C, F; very simple, subrectangular in lateral view, with denticles on anterior flagellum-conducting lamella (Figure 4C).

*Vulvae* (Figure 5A–B): Vulvae typical of the *C.perforatus*-group: glabrous operculum, bursa with a few setae and the receptaculum seminis as a stalked sphere with a small tubular appendix.

####### Etymology.

The new species is dedicated to the VILLUM Foundation, named after Villum Kann Rasmussen (1909–1993), as recognition for the generous support to research in natural sciences.

####### Distribution.

*Cylindroiulusvillumi* sp. n. was discovered in the cave Algar do Pena, located in the Santo António plateau, the central sub-unit of the Estremenho karst massif in central Portugal. It was found inside a big piece of deadwood located at the base of the entrance pit to the cave, at a depth of 33 meters below the surface.

####### Ecology.

Algar do Pena is the largest underground chamber of Portugal. The temperature is very constant 13 ±1 °C and relative humidity close to saturation. It is a very oligotrophic cave where only a few cave-adapted species are recorded: the spider *Nesticuslusitanicus* Fage, 1931, the terrestrial isopod *Trichoniscoidesmeridionalis* Vandel, 1946, the springtail *Onychiurusconfugiens* Gama, 1962; the dipluran Podocampacf.fragiloides Silvestri, 1932; and the beetle *Trechusgamae* Reboleira & Serrano, 2009 (Reboleira 2007, 2012, Reboleira and Ortuño 2011, Reboleira et al. 2009, 2010, 2011). The holotype and a juvenile male paratype have ‘Amphoromorpha’ fungi on the head and antenna, similar to those observed by Enghoff and Reboleira (2017) on other millipedes.

## Discussion

The genus *Cylindroiulus* exhibits great morphological diversity concerning size, ocelli, number of segments, pigmentation, chaetotaxy, legs, accessory claws, and genital structures (Reboleira and Enghoff 2014c, Read 1989a, b); in the endemic *C.madeirae*-group from Madeira Island, part of the diversity can be seen as a result of niche segregation (Enghoff 1982, 1983, 2011). The new species *C.villumi* is the first representative of the genus with an exclusively subterranean life-style and corresponding troglomorphic characters (i.e., unpigmented, anophthalmic, elongated sensory cones on the antenna, and very short accessory claw) found in continental Europe. The new species can be distinguished from all congeners by the combination of anophthalmy and the shape of the male gonopods. The extremely short accessory claws, a trait believed to be associated with the ability to climb (Enghoff 1983), has also been observed in the other cave-adapted species of the genus (Reboleira and Enghoff 2014c).

Two anophthalmic, subterranean-adapted species of *Cylindroiulus*, *C.julesvernei* and *C.oromii*, are known from caves of Madeira Island (Reboleira and Enghoff 2014c). From the rest of the vast distribution area of *Cylindroiulus*, only two anophthalmic species are known: *C.vulnerarius* (Berlese, 1888), a species that has occasionally been found in caves but which is quite widespread in epigean soil habitats in West Europe (Schubart 1934, Kime and Enghoff 2017), and *C.gregoryi* Read, 2007 from the Galician province of Spain.

*Cylindroiulusvillumi* sp. n. belongs to the nominal subgenus Aneuloboiulus Verhoeff, 1899 which is characterized by traits regarded as plesiomorphic (Enghoff 1982, Read 2007). Within this assemblage, *C.villumi* sp. n. may, with some uncertainty, be placed in the Iberian *C.perforatus*-group. This group, which contains four Iberian species, is characterized by a “window”, usually a perforation, in the gonopodal promerite; in other characters, the group is similar to the endemic Macaronesian *C.madeira*-group, notably by the presence of denticles on the flagellum-conducting lamella of the solenomerite, the naked vulvar operculum and the tubular appendix to the receptaculum seminis (Read 1989a, b, 2007). *Cylindroiulusvillumi* does not have a perforated promite, but it does have denticles on the anterior flagellum-conducting lamella on the gonopod solenomerite like species of the *C.perforatus*-group; also the vulva of *C.villumi* sp. n. with the glabrous operculum and the subspherical receptaculum seminis with a small terminal appendix are typical of the *C.perforatus*-group. In these characters our new species is also similar to the Macaronesian *C.madeirae*-group (Enghoff 1982), presumably the sister-group of the *C.perforatus*-group (Read 1989a, b); however, the pilosity of the preanal ring in *C.villumi* sp. n. more resembles that of the *C.perforatus*-group.

The Algar do Pena Cave, where the new species was found, has been intensively sampled over the last decade, and only very recently this species was collected (Reboleira 2007, 2012). Several other caves in the same massif have been intensively sampled without retrieving any specimen of the new species. Only the troglophile chordeumatidan *Haplobainosomalusitanum* Verhoeff, 1900 (family Haplobainosomatidae) has been recorded in caves of the same massif, while two epigean unidentified species of *Cylindroiulus* are also known from the mesovoid shallow substrate (MSS) in same massif (Reboleira and Enghoff 2014a).

## Supplementary Material

XML Treatment for Cylindroiulus villumi
